# Beyond endocrine features in non-classical congenital adrenal hyperplasia: a narrative review of psychoneuro-social perspectives in pediatric and adolescent patients

**DOI:** 10.1007/s00431-025-06673-w

**Published:** 2025-12-13

**Authors:** Roberto Paparella, Fabiola Panvino, Ida Pucarelli, Marcello Niceta, Matteo Spaziani, Alberto Spalice, Francesco Pisani, Ignazio Ardizzone, Luigi Tarani

**Affiliations:** 1 Department of Maternal Infantile and Urological Sciences, Sapienza University of Rome, Rome, Italy; 2 Department of Human Neuroscience, Section of Child and Adolescent Neuropsychiatry, Sapienza University of Rome, Rome, Italy; 3 Department of Molecular Genetics and Functional Genomics, Ospedale Pediatrico Bambino Gesù, IRCCS, Rome, Italy; 4 Department of Experimental Medicine, Sapienza University of Rome, Rome, Italy; 5 Department of Theoretical and Applied Sciences, eCampus University, Novedrate, Italy

**Keywords:** Non-classical congenital adrenal hyperplasia, 21-hydroxylase deficiency, *CYP21A2*, Heterozygous carriers, Pediatric endocrinology, Psychoneuro-social outcomes, Quality of life

## Abstract

Non-classical congenital adrenal hyperplasia (NCCAH) is a late-onset, milder form of 21-hydroxylase deficiency marked by adrenal androgen excess. While endocrine aspects are well described, psychoneuro-social outcomes in pediatric and adolescent patients remain less clearly defined. A literature search of MEDLINE (PubMed) was conducted in July 2025 using “Non-Classical Congenital Adrenal Hyperplasia” with terms related to mental health, cognition, social adaptation, gender identity, sexual orientation, and heterozygous carriers. In females, androgen excess is frequently associated with anxiety, depression, body image concerns, and increased prevalence of gender nonconformity or non-heterosexual orientation, although most maintain a female gender identity. Males generally present fewer gender identity concerns, with inconsistent evidence for higher externalizing behaviors. Neurocognitive outcomes are variable, with occasional subtle deficits in working memory, executive function, or attention, alongside possible strengths in visuospatial tasks. Health-related quality of life in adequately treated pediatric NCCAH is generally comparable to that of healthy peers, though age-specific psychosocial vulnerabilities may occur. Social difficulties, particularly among females, may involve stigmatization and reduced peer acceptance. Heterozygous *CYP21A2* carriers are usually asymptomatic and do not exhibit significant psychoneuro-social burden; interventions are not routinely indicated unless hyperandrogenic symptoms are present. *Conclusions*: Psychoneuro-social effects of NCCAH are heterogeneous and shaped by sex, age, and clinical presentation. An individualized, multidisciplinary approach integrating endocrinology, psychological support, and social resources is recommended. Longitudinal studies are needed to clarify long-term trajectories and optimize targeted interventions.

**Table:** 

**What is Known:** • *NCCAH is a common form of CAH with mild androgen excess.* • *Its endocrine features are well described, but psychosocial aspects are often overlooked.* **What is New:** • *This review highlights cognitive, emotional, and social challenges in pediatric NCCAH.* • *Sex-specific patterns and the role of early diagnosis in improving outcomes are emphasized.*

**Supplementary information:**

The online version contains supplementary material available at 10.1007/s00431-025-06673-w.

## Introduction

Congenital adrenal hyperplasia (CAH) encompasses a group of autosomal recessive disorders resulting from enzyme deficiencies that impair cortisol biosynthesis in the adrenal glands [1]. The most common form, 21-hydroxylase deficiency, accounts for approximately 90–95% of CAH cases and can present in classical or non-classical forms depending on the severity of enzymatic impairment [2]. While classical CAH may manifest in infancy with adrenal insufficiency in both sexes and ambiguous genitalia in affected females, non-classical CAH (NCCAH) has a milder phenotype and typically presents later in childhood, adolescence, or even adulthood. NCCAH is often diagnosed incidentally or during the evaluation of symptoms such as premature pubarche, hirsutism, menstrual irregularities, or unexplained infertility in adults [3].

NCCAH is caused by mutations in the *CYP21A2* gene, leading to partial enzyme deficiency and consequent androgen excess. Unlike classical CAH, individuals with NCCAH usually retain sufficient cortisol production to prevent adrenal crises, but they may experience significant androgen-driven effects that impact growth, pubertal development, and metabolic health. In females, symptoms such as hirsutism, acne, menstrual disturbances, and virilization can affect self-esteem and social interactions [4]. Males with NCCAH may exhibit subtle symptoms such as early beard growth, accelerated bone age, or subclinical testicular adrenal rest tumors that may contribute to future fertility concerns [5].

The prevalence of NCCAH varies by ethnicity and population genetics, with estimates ranging from 1 in 200 to 1 in 1000 individuals in Caucasian populations, though it is more common in certain ethnic groups such as Ashkenazi Jews, where the carrier frequency is significantly higher [6]. Given its autosomal recessive inheritance, many individuals may be asymptomatic heterozygous carriers, raising questions about potential subclinical effects on psychological and social well-being even in those without overt NCCAH symptoms [7].

From a psychological perspective, NCCAH presents unique challenges, particularly in females, where androgen excess can lead to concerns related to body image, gender identity, and emotional well-being. Studies have suggested that individuals with NCCAH may be at increased risk for anxiety, depression, and social withdrawal, potentially due to the interplay between hormone exposure and psychosocial stressors [8]. Furthermore, the impact of androgens on brain development raises questions regarding potential neurocognitive differences in affected individuals, including alterations in executive functioning, attention, and behavior [9].

Socially, children and adolescents with NCCAH may face difficulties related to gender expectations, peer interactions, and self-perception. Early androgen exposure can influence play preferences, career interests, and even sexual orientation in some cases, contributing to a complex psychosocial profile [10]. Additionally, the visible physical manifestations of androgen excess, such as hirsutism and acne, may lead to stigmatization, bullying, and reduced self-confidence, particularly in adolescent females [11]. In males, while increased androgens may confer some advantages in terms of social dominance and competitive behaviors, the long-term psychosocial implications remain less well studied [12].

Another important consideration is the experience of heterozygous carriers of *CYP21A2* mutations. While traditionally considered asymptomatic, emerging evidence suggests that some carriers may exhibit subtle endocrine and metabolic differences, raising the possibility of minor psychological or neurodevelopmental effects [13]. Carriers may have slightly altered androgen levels, which could impact mood regulation, stress response, and cognitive processing [14].

Understanding these nuances is critical for a comprehensive approach to NCCAH and related conditions. This review aims to synthesize available evidence on the psychological, neurocognitive, and social functioning of children and adolescents with NCCAH, and to identify potential risk and protective factors across biological and psychosocial domains.

## Methods

We performed a narrative review of MEDLINE (PubMed) in July 2025 using the query: (“non-classical congenital adrenal hyperplasia” OR “NCCAH” OR “nonclassic CAH”) AND (psych* OR mental OR anxiety OR depression OR cognition OR neurocognitive OR “executive function” OR social OR “quality of life” OR “gender identity” OR “sexual orientation” OR “heterozygous”). We focused on pediatric and adolescent populations (0–18 years) and included studies reporting psychoneuro-social outcomes. Reference lists of key reviews were hand-searched. Data were qualitatively synthesized due to heterogeneity of designs and outcomes. The main characteristics of the studies assessing psychoneuro-social outcomes included in this review are summarized in Supplementary Table S1. Limitations include potential publication bias and variability in diagnostic criteria for NCCAH and carrier status. By addressing the psychological, cognitive, and social dimensions of NCCAH, this work seeks to provide a holistic understanding of the condition and inform clinical approaches to improving patient outcomes (Table 1).

**Table 1:** Proposed psychosocial care recommendations for pediatric and adolescent patients with non-classical congenital adrenal hyperplasia (NCCAH)

Recommendation	Rationale	Suggested implementation
Early involvement of mental health professionals with expertise in disorders of sex development (DSDs)	Ensures timely identification of psychological, social, and family concerns; promotes holistic management	Referral to psychologists or psychiatrists familiar with DSDs soon after diagnosis
Psychoeducation for patients and families	Enhances disease understanding, reduces anxiety, and promotes adherence	Age-appropriate education sessions addressing NCCAH pathophysiology, symptoms, and management
Individual and family counseling	Addresses body image concerns, stigma, and emotional distress from androgen excess	Sessions focused on coping strategies for visible symptoms (e.g., hirsutism, acne, menstrual irregularities, premature pubarche, gynecomastia)
Support for coping with chronic illness	Improves resilience and self-esteem; mitigates school and peer relationship challenges	Development of personalized coping plans and collaboration with school personnel
Facilitation of peer support and patient/family networks	Reduces isolation, fosters shared experiences and mutual support	Connection to patient advocacy groups, moderated peer meetings, or online communities
Routine psychosocial screening	Enables early detection of mental health or adjustment problems	Use of validated screening tools at regular intervals during follow-up
Low threshold for psychological or psychiatric referral	Prevents escalation of mental health concerns; ensures early treatment	Prompt referral when screening indicates emerging symptoms or when family expresses concern
Interdisciplinary care model	Integrates endocrinology, psychology, and social support for optimal outcomes	Regular multidisciplinary team meetings and coordinated care planning

## Psychological implications, neurocognitive outcomes, social adaptation, and quality of life in NCCAH

### Mental health disorders

Several studies and registry-based data have shown that females with NCCAH have an increased prevalence of psychiatric disorders, particularly stress-related and adjustment disorders, as well as alcohol use disorders. This risk appears more pronounced in individuals with more severe genotypes, yet remains elevated in NCCAH patients compared to unaffected controls [2, 15]. Clinical signs of androgen excess—such as hirsutism, acne, and voice deepening—have been linked to negative body image, social withdrawal, and avoidance of romantic or sexual relationships, in addition to a heightened risk of depressive and anxiety symptoms [1, 16, 17]. The Endocrine Society has emphasized that diminished quality of life and greater psychosocial burden are more common in females with CAH, including the non-classical form, than in the general population [2].

In males with NCCAH, evidence of increased externalizing behaviors—such as impulsivity, aggression, hyperactivity, risk-taking, and conduct-related problems—is limited and less consistent than in females. The majority of males with NCCAH are asymptomatic, and existing studies do not consistently report a significant increase in these behaviors when compared to control groups. However, some data suggest the presence of cognitive complaints and higher levels of autistic traits in males with CAH as a broader category [16, 18]. Currently, the literature does not clearly support a causal relationship between androgen-driven neuroendocrine alterations and externalizing behavioral outcomes in males with NCCAH.

### Gender identity and sexual orientation

Females with NCCAH demonstrate a higher frequency of gender nonconforming behaviors, a modestly elevated—though still overall low—incidence of gender dysphoria, and an increased prevalence of same-sex attraction when compared to the general female population. These patterns are believed to result from mild prenatal androgen exposure, which is thought to influence gender-typical behaviors and aspects of sexual orientation in a dose-dependent manner. Empirical evidence consistently indicates that females with NCCAH tend to exhibit more traditionally male-typed preferences and behaviors, including interests aligned with male-dominated careers and recreational activities. Additionally, they are more likely to report non-heterosexual orientation compared to unaffected peers, although the majority continue to identify as both female and heterosexual [19–24]. Importantly, despite the observed behavioral variations, the prevalence of gender dysphoria remains low, and most affected individuals retain a female gender identity [2].

Conversely, males with NCCAH do not appear to diverge significantly from unaffected males in terms of gender identity or sexual orientation. Current evidence suggests that prenatal and postnatal androgen exposure in these individuals does not correlate with increased rates of gender nonconformity or same-sex attraction [17, 25].

### Cognitive function and executive processing

While classic forms of CAH have been associated with a masculinized cognitive profile—most notably enhanced visuospatial abilities—females with NCCAH do not consistently exhibit this cognitive shift. In contrast, some studies suggest a relative advantage in domains typically favoring females, such as episodic memory and emotion recognition, rather than in spatial performance [26].

Neuropsychological assessments in individuals with CAH, including those with milder phenotypes, have identified specific impairments—when present—in areas such as visual working memory, executive functioning, and attentional control, whereas general intellectual ability and verbal skills tend to remain preserved [27–29]. Although several of these studies included mixed cohorts or predominantly classical CAH, their applicability to NCCAH remains limited, and findings should therefore be interpreted cautiously. Adolescent females with CAH, including NCCAH, have been reported to perform less well than unaffected peers on tasks assessing visual memory span and free recall, with delayed diagnosis correlating with poorer memory performance [28]. Neuroimaging studies have revealed structural alterations in cortical regions implicated in executive functioning and working memory, particularly the prefrontal and parietal cortices, which may provide a neural basis for these observations [29, 30]. Nevertheless, the degree of impairment is typically mild and marked by considerable interindividual variability. The “brain fog” or reduced attentional efficiency occasionally reported in females with NCCAH may partly reflect secondary psychological distress rather than direct androgenic effects, as this symptom is rarely described in males.

Cognitive outcomes in NCCAH are modulated by several factors, including disease severity, age at diagnosis, cumulative exposure to glucocorticoid therapy, as well as educational, social, and environmental influences [27, 28, 31]. Earlier diagnosis and appropriately tailored medical management are generally associated with more favorable neurocognitive profiles [31]. Deficits in executive functioning and working memory may contribute to academic difficulties and hinder social adaptation, thereby amplifying the psychological burden already documented in females with NCCAH—such as mood disturbances, anxiety, and body image dissatisfaction. In contrast, current data do not demonstrate consistent cognitive differences in males with NCCAH.

Taken together, available studies reveal inconsistent findings regarding cognitive performance in NCCAH, reflecting significant methodological heterogeneity. Variability in diagnostic criteria, age at diagnosis, hormonal control, and treatment regimens—along with differences in test batteries and small sample sizes—likely contribute to divergent results. Socioeconomic background, parental education, and comorbidities may further confound these associations. Overall, the evidence supports normal cognitive functioning in most patients but highlights the need for standardized, longitudinal assessments.

### Neurodevelopmental and mood disorders

There is no robust evidence for an increased prevalence of neurodevelopmental disorders such as attention-deficit/hyperactivity disorder (ADHD) in individuals with NCCAH, particularly in males. Large-scale cohort and registry-based studies have not consistently demonstrated a significant rise in ADHD or related neurodevelopmental disorders among males with NCCAH when compared to control populations. Nevertheless, certain subgroups of males with CAH, particularly those diagnosed later in life, have shown elevated rates of psychiatric comorbidities, including psychotic disorders and substance use disorders [18, 32]. Additionally, a recent Mendelian randomization analysis has suggested a potential causal link between *CYP21A2* deficiency and increased risk of autism spectrum disorder; however, this association is not specific to NCCAH and requires further investigation and replication in targeted studies [33].

With respect to mood disorders, the current body of evidence does not indicate a consistent elevation in conditions such as dysthymia or bipolar spectrum disorders in individuals with NCCAH. Instead, the literature more frequently reports increased rates of anxiety, depression, and adjustment-related psychopathologies, particularly among females. Proposed pathophysiological mechanisms include chronic androgen excess and impaired cortisol biosynthesis, which may influence central dopaminergic and serotonergic pathways, thereby affecting mood regulation, executive functioning, and attentional processes [1, 34–37]. However, direct and specific associations between these hormonal alterations and defined neurodevelopmental or mood disorders in NCCAH remain hypothetical and unconfirmed by robust empirical data. Moreover, most data on psychiatric comorbidities derive from classical CAH or unstratified cohorts; evidence specific to NCCAH is still scarce and requires confirmation in well-defined pediatric samples. The heterogeneity of reported emotional outcomes may arise from multiple factors, including variation in disease severity, hormonal control, and age at diagnosis. Differences in glucocorticoid treatment duration, psychological assessment tools, and sociocultural context can also influence results. Consequently, comparisons across studies are challenging, and findings should be interpreted cautiously.

### Peer relationships and social competence

Hormonal imbalances and physical manifestations associated with NCCAH can adversely affect peer interactions and social functioning during childhood and adolescence, with a more pronounced impact observed in females. Androgen-related physical features—such as hirsutism, acne, and increased muscularity—are common in females with NCCAH and are frequently linked to experiences of teasing, peer rejection, and social marginalization. These physical differences can contribute to negative body image, diminished self-esteem, and difficulties initiating or sustaining friendships, as well as avoidance of social engagement and intimate relationships [10, 17, 38].

Males with NCCAH may exhibit certain socially advantageous traits—such as increased self-confidence or assertiveness—potentially related to early androgen exposure and early puberty. However, these potential benefits may be offset by tendencies toward impulsivity, heightened risk-taking, or difficulties aligning socially with age-matched peers. Despite these observations, current evidence suggests that the majority of affected males are asymptomatic, and consistent findings regarding social advantages or disadvantages are lacking [1, 17]. These observations are largely based on studies including both classical and non-classical forms of CAH. Consequently, caution is warranted when generalizing such findings to the NCCAH population.

Concerns about gender expression and societal expectations can further complicate social interactions for both sexes. Issues related to gender expression and societal norms may further complicate peer relationships in both sexes. Females with NCCAH often show preferences for male-typical interests and activities, which can lead to social friction in contexts where rigid gender roles are enforced [2]. Psychosocial support systems—including parental involvement, inclusive school environments, and targeted educational initiatives—play a key role in addressing these challenges. Interventions such as family counseling, anti-bullying programs, and psychoeducation for educators and peers have been shown to foster resilience, promote social inclusion, and enhance overall social competence in affected children and adolescents [34, 38, 39]. Conflicting results regarding social adjustment likely reflect differences in study design, sample composition, and cultural expectations surrounding gender roles and chronic disease. Many studies relied on parental reports rather than self-assessment, introducing potential reporting bias. Future research should use validated age-specific questionnaires and consider both biological and contextual moderators of social adaptation.

### Academic and career implications

Educational outcomes in individuals with NCCAH are often adversely influenced by psychosocial challenges, particularly among females. Factors such as diminished self-confidence, heightened social anxiety, and body image dissatisfaction may contribute to reduced school participation and suboptimal academic performance [38–40]. The chronic course of NCCAH—with its requirement for lifelong monitoring and potential hormonal instability—can further impair attention, motivation, and daily functioning. In parallel, the higher prevalence of mood disorders, including anxiety and depression, may exacerbate school absenteeism and hinder academic attainment [38–40].

Cognitive domains potentially modulated by androgen exposure, such as visuospatial processing, do not consistently show enhancement in females with NCCAH. In fact, some evidence suggests a relative advantage in abilities typically favoring females, such as episodic memory and emotional recognition, rather than improvements in spatial skills. Moreover, any putative cognitive benefit is often counterbalanced by impairments in executive functioning and working memory, which can negatively impact academic performance—particularly in cognitively demanding environments. Nonetheless, a subset of individuals may demonstrate aptitude in spatial reasoning and problem-solving, potentially facilitating engagement in STEM (science, technology, engineering, and mathematics) fields, although this is not uniformly observed [26, 41].

Long-term career outcomes are shaped by complex interactions between self-perception, sociocultural expectations, and potential workplace biases linked to visible endocrine-related features. Females with NCCAH may face reduced self-efficacy and altered career trajectories due to stigma, gender nonconformity, or concerns regarding physical appearance, potentially restricting access to certain professions or limiting professional advancement [10, 40]. Epidemiological data indicate that women with milder CAH genotypes (including NCCAH) are more likely to attain higher educational qualifications, yet less likely to marry or have children, and may exhibit elevated rates of sick leave or disability benefit use—reflecting persistent psychosocial and health-related burdens [40].

### Quality of life

Evidence on health-related quality of life (HRQoL) in children and adolescents with NCCAH is limited, with only a few studies employing validated instruments such as the Pediatric Quality of Life Inventory (PedsQL™). In a cross-sectional case–control study of 23 hydrocortisone-treated patients (mean age range spanning childhood to adolescence), HRQoL scores reported by both participants and parents were comparable to those of healthy siblings, with no significant differences in total or domain scores. Anthropometric measures were within the normal range, and in NCCAH subjects, higher body mass index standard deviation scores correlated positively with total, school functioning, and psychosocial HRQoL scores, suggesting a possible influence of body composition on perceived well-being [39]. Similarly, a Portuguese single-center study including 19 patients (2–18 years old) with NCCAH found that HRQoL was generally preserved across most age groups. The only significant differences emerged in parent-proxy reports for children aged 8–12 years, where physical and emotional health scores were higher than controls, but psychosocial health and total scale scores were lower [42]. These findings suggest that, in adequately treated pediatric NCCAH populations, HRQoL is largely comparable to that of healthy peers, although specific age-related psychosocial vulnerabilities may exist, particularly in preadolescence. Both studies highlight the need for larger, longitudinal investigations to better define potential subtle impacts on HRQoL and to identify subgroups that might benefit from targeted psychosocial support.

### Mechanistic pathways linking hormonal imbalance to psychoneuro-social outcomes

**Fig. 1:**
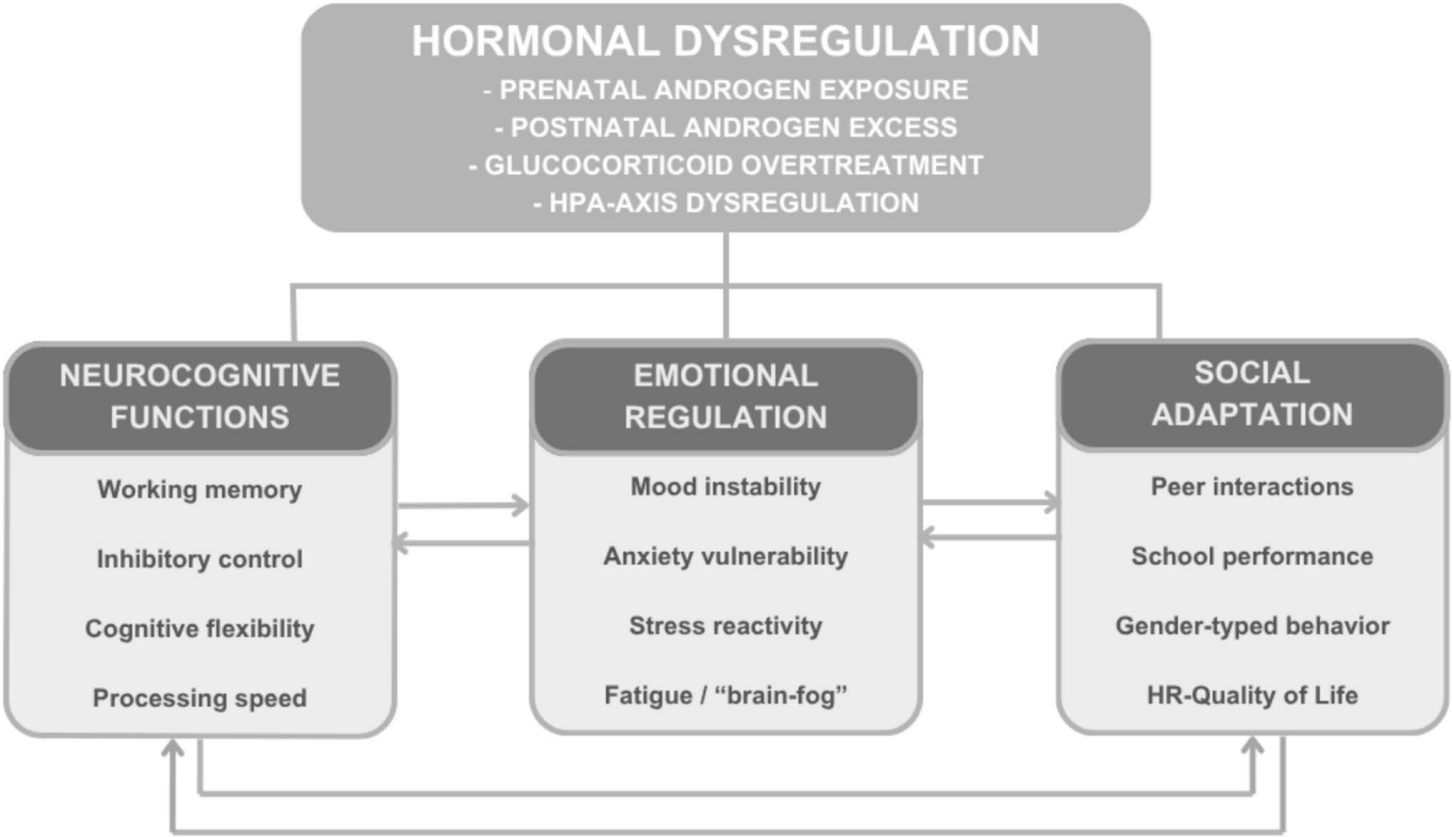
Integrated psycho-neuro-social model of pediatric NCCAH

Several mechanisms may underlie the association between hormonal dysregulation and psychoneuro-social outcomes in NCCAH (Fig. 1). Across the various domains, apparent inconsistencies among studies should be viewed in light of methodological differences, diagnostic variability, and uncontrolled confounders. Recognizing these sources of heterogeneity provides a more coherent conceptual framework for interpreting the current evidence regarding NCCAH.

Altered prenatal androgen exposure is proposed to influence brain sexual differentiation and early programming of cognitive and emotional circuits, as demonstrated by studies in classic CAH showing masculinization of play behavior and interests in affected females, with meta-analytic evidence for robust effects on sex-typical play [43]. However, neuroimaging studies have not consistently identified corresponding structural changes, except for some findings in emotion processing and reduced white matter integrity, suggesting disease-related rather than purely androgen-driven mechanisms [44]. Postnatal androgen excess may modulate reward processing and social behavior via dopaminergic and limbic pathways, but direct evidence in non-classic CAH is sparse. Subtle dysregulation of the hypothalamic–pituitary–adrenal (HPA) axis is present even in mild forms and may contribute to psychiatric vulnerability [1]. Chronic glucocorticoid overtreatment, as seen in classic CAH, is associated with impaired hippocampal and prefrontal function, reduced white matter integrity, and cognitive deficits such as working memory impairment and “brain-fog” complaints [30]. These effects are dose-dependent and may be relevant in non-classic CAH if overtreatment occurs. Psychosocial stress and inflammatory mediators may interact with HPA axis dysregulation, amplifying emotional vulnerability, but this remains speculative and requires further longitudinal, multimodal studies integrating hormonal, neuroimaging, and psychological data [1, 30, 44]. Overall, while these mechanisms are plausible, the evidence base in non-classic CAH is limited, and further research is needed to clarify causal pathways and clinical relevance (Table 2).

**Table 2:** Current evidence gaps and future research priorities in pediatric and adolescent NCCAH

Area	Main evidence gap	Research priority
Diagnostic definitions	Inconsistent criteria for NCCAH and carrier status across studies	Standardized genotype–phenotype classification
Study design	Predominance of small, cross-sectional, single-center cohorts	Multicenter longitudinal studies with harmonized outcomes
Cognitive outcomes	Heterogeneous tests and lack of normative pediatric data	Use of validated neurocognitive batteries and age-matched control
Emotional/psychiatric outcomes	Limited assessment of anxiety, depression, and stress coping	Integration of standardized psychological questionnaires
Sex-specific analyses	Insufficient attention to male cohorts	Balanced recruitment and stratified analyses by sex
HRQoL measures	Different instruments and informants used	Consensus on core HRQoL measures for pediatric CAH
Carriers	Sparse and inconsistent data	Prospective studies on psychosocial adaptation in carriers
Mechanistic studies	Limited exploration of neuroendocrine pathways	Multimodal studies combining hormonal, neuroimaging, and psychological assessments

### Sex-specific differences in psychosocial outcomes

Sex-related variations described across studies likely arise from an interplay of biological, clinical, and sociocultural influences. Females with NCCAH more frequently experience visible hyperandrogenic features—such as hirsutism, acne, and menstrual irregularities—that can adversely affect body image and self-esteem, particularly during adolescence [17]. In contrast, males often exhibit milder or subclinical phenotypes and may remain undiagnosed, facing fewer visible stressors but potentially different psychosocial trajectories [17]. Differences in androgen receptor sensitivity, timing and duration of hormonal exposure, and cultural gender norms may further modulate these outcomes [17, 19, 45]. Understanding these sex-specific mechanisms is crucial for tailoring psychosocial support and clinical management strategies in pediatric NCCAH [2].

## Heterozygous carriers of *CYP21A2* mutations

Individuals heterozygous for *CYP21A2* mutations—i.e., carrying a single pathogenic allele—are generally considered phenotypically unaffected and do not meet the clinical or biochemical criteria for either NCCAH or the classical form of CAH. Nonetheless, emerging evidence suggests that a subset of heterozygous carriers, particularly females, may exhibit mild hyperandrogenic manifestations, including hirsutism, menstrual irregularities such as oligomenorrhea, or clinical features overlapping with polycystic ovary syndrome (PCOS), especially during adolescence. The expression and severity of these features may be influenced by the specific mutation involved and possibly by the presence of regulatory polymorphisms affecting gene expression [13, 46].

Evidence regarding heterozygous *CYP21A2* carriers remains limited. Although mild clinical signs may be present in some carriers, the overall psychoneuro-social burden is markedly lower than that observed in individuals with biallelic pathogenic variants (Table 3). Most pediatric carriers are asymptomatic; however, some adolescents—particularly females with mild hyperandrogenic features—may report lower self-esteem or body-image dissatisfaction. Current data do not indicate significant cognitive or emotional impairment. Nevertheless, clinicians should consider psychological support on an individual basis when clinical symptoms or psychosocial distress arise [13, 46, 47].

**Table 3:** Summary of main characteristics in pediatric and adolescent patients with non-classical congenital adrenal hyperplasia (NCCAH) and heterozygous carriers of *CYP21A2* mutations

	Females with NCCAH	Males with NCCAH	Heterozygous ***CYP21A2*** carriers
Clinical onset and androgen excess	Often present in late childhood or adolescence; androgen excess common with hirsutism, acne, menstrual irregularities [17]	Often asymptomatic or detected via family screening; androgen excess usually mild to moderate [17]	Usually asymptomatic; rare mild hyperandrogenic features (e.g., hirsutism, menstrual irregularities in females) [13, 46, 47]
Psychological aspects	Increased prevalence of anxiety, depression, body image concerns; higher rates of gender nonconformity and non-heterosexual orientation, though female gender identity is usually maintained [17, 48, 49]	Generally fewer gender identity-related concerns; inconsistent evidence for higher impulsivity or externalizing behaviors [17, 32, 50]	No consistent evidence of increased psychiatric vulnerability; psychosocial burden minimal [13, 46, 47]
Neurocognitive outcomes	Subtle deficits in working memory or executive function; possible strengths in visuospatial skills [27, 28, 30]	Variable findings; occasional attention regulation difficulties or attention-deficit/hyperactivity disorder diagnosis [17, 32, 50]	Generally normal; isolated reports of mild mood or cognitive differences [13, 46, 47]
Social effects	Stigmatization due to physical signs; potential peer rejection; reduced self-esteem [2, 38]	Social confidence may be increased, but risk-taking behaviors can occur [17, 50]	Social functioning typically unaffected; occasional body image concerns in symptomatic carriers [13, 46, 47]

Information is based on available pediatric/adolescent data; evidence remains limited for some outcomes, and findings may vary by sex, age, and treatment status. Routine psychosocial interventions are not recommended for asymptomatic carriers

## Conclusion

The psychoneuro-social profile of NCCAH in pediatric and adolescent patients is shaped by a complex interplay of biological, developmental, and environmental factors. While some females experience anxiety, depression, body image concerns, and gender nonconformity, many—especially when adequately treated—show preserved quality of life and social functioning. Males generally present fewer psychosocial or neurocognitive difficulties, though occasional externalizing behaviors are reported. Cognitive outcomes are variable and typically involve subtle deficits in working memory or executive processes, with early diagnosis and optimized treatment associated with better results. HRQoL studies indicate overall preservation, with isolated vulnerabilities in specific age groups. Overtreatment should be carefully avoided, as supraphysiological glucocorticoid exposure may exacerbate fatigue, mood fluctuations, and cognitive complaints. Regular dose adjustment—often to lower levels than required in simple virilizing CAH—is essential for psychological as well as somatic well-being. Heterozygous *CYP21A2* carriers seldom manifest clinically relevant psychoneuro-social issues, and targeted interventions are rarely necessary unless overt hyperandrogenism is present. Given this heterogeneity, care models should be individualized, combining endocrinological management with psychological and social support when indicated. Longitudinal, multicenter studies are essential to delineate long-term neurocognitive and psychosocial outcomes and to inform tailored intervention strategies. Future research should specifically focus on NCCAH populations, as current understanding of psychological and neurocognitive outcomes often relies on extrapolations from classical or mixed CAH cohort.

## Supplementary information

Below is the link to the electronic supplementary material.

ESM 1 (DOCX 42.5 KB)

## Data Availability

All data supporting the conclusions of this review are included within the article and its supplementary material. No additional datasets were generated or analyzed for this study.

## Abbreviations

ADHD: Attention-deficit/hyperactivity disorder
CAH: Congenital adrenal hyperplasia
DSDs: Disorders of sex development
HPA: Hypothalamic-pituitary-adrenal
HRQoL: Health-related quality of life
NCCAH: Non-classical congenital adrenal hyperplasia
PCOS: Polycystic ovary syndrome
PedsQL™: Pediatric Quality of Life Inventory
