# Supplementary material for: Beyond endocrine features in non-classical congenital adrenal hyperplasia: a narrative review of psychoneuro-social perspectives in pediatric and adolescent patients
Source: Eur J Pediatr. 2025 Dec 13;185(1):11. doi: 10.1007/s00431-025-06673-w (PMC12701048; doi:10.1007/s00431-025-06673-w)
Supplement: Supplementary file 1 — (DOCX 42.5 KB) [file 431_2025_6673_MOESM1_ESM.docx]

# Supplementary Table S1. Characteristics of included research studies assessing neuropsychological aspects in CAH.

| No. | Author (Year) | Country | Study design | Sample size | Sex distribution (cases) | Age range (cases) | Main findings |
| --- | --- | --- | --- | --- | --- | --- | --- |
| 5 | Nandagopal et al (2011) | USA | Cross-sectional family-genetic profiling study | 249 parents (145 unrelated CAH families) | 10 (7 females and 3 males) were found to have cryptic NCCAH | Adult: full age range not explicitly given | “Cryptic NCCAH” parents are mostly asymptomatic (clinical and hormonal); female infertility common, no clinical neuropsychological findings, no need for routine therapy in absence of symptoms. |
| 13 | Guarnotta et al (2004) | Italy | Cross-sectional observational study | 57 females with *CYP21A2* variants (24 NCCAH, 33 heterozygous carriers) vs 44 healthy female controls | 57 F | Adolescent and adult women: full age range not explicitly given | Heterozygous carriers of *CYP21A2* mutations (monoallelic state) showed a high prevalence of late-onset signs of hyperandrogenism and polycystic ovary syndrome–like phenotype (hirsutism, oligomenorrhea, overweight) compared with controls; hormonal indices (17-OHP/cortisol ratio) differed between carriers and NCCAH |
| 14 | Charmandari et al (2004) | USA | Observational case–control study | 18 carriers (parents of children with classic CAH) vs 16 controls | 6 M / 12 F | 30.2–50.1 y | Heterozygous carriers of 21-OH deficiency exhibit mild hypocortisolism and compensatory CRH hypersecretion, suggesting a predisposition to stress-related vulnerability rather than overt psychopathology. |
| 15 | Engberg et al (2015) | Sweden | Nationwide population-based cohort study | 335 females with CAH vs 33,500 female controls and 33,500 male controls | 335 F | Born between 1915 and 2010 (age range not provided) | Females with CAH showed significantly higher odds of any psychiatric diagnosis. The risk of alcohol misuse was markedly higher and was highest in those with the most severe “null” genotype. The risk of stress/adjustment disorders was also about doubled vs female controls. |
| 16 | Lašaitė et al (2022) | Lithuania | Cross-sectional case–control study | 26 CAH patients vs 26 controls | 11 M / 15 F | 14–48 y | Women with CAH reported higher perceived distress, tension-anxiety, depression-dejection, fatigue-inertia, and worse environmental domain of quality of life compared to age-matched control women. Men with CAH did not differ significantly from age-matched male controls. |
| 18 | Ridder et al (2024) | Denmark | Observational cross-sectional case–control study | 37 CAH patients vs 33 controls | 11 M / 26 F | 18 – 70 years (eligible range);  mean age ± SD = 35.4 ± 11.0 (females) and 31.4 ± 8.1 (males) | Adults with CAH exhibited a sex-specific comorbidity profile: females with CAH showed decreased height, higher BMI and hemoglobin, reduced insulin sensitivity, cardiac diastolic dysfunction, and lower self-reported quality of life compared to female controls; males with CAH reported more cognitive complaints and higher autistic trait scores compared to male controls |
| 19 | Segev-Becker et al (2020) | Israel | Prospective questionnaire-based case-control study | 38 NCCAH vs 62 controls | Female | 18-44 y | Adult women with NCCAH did not differ significantly from controls in health-related quality of life, gender identity, or sexuality. Subtle variations (e.g., occasional non-binary feelings, first love with a woman) could indicate a continuum of androgen influence extending from normal to mild (NCCAH) to severe (classic CAH). |
| 20 | Frisén et al (2009) | Sweden | Cross-sectional case-control study | 62 women with CAH (NC = 5) vs 62 controls | Female | 18-63 y | Women with CAH showed more male-typical occupational and leisure interests, higher rates of non-heterosexual orientation, and delayed sexual debut compared with controls. The degree of gender-atypical behavior correlated with genotype severity. Despite overall normal quality of life, many reported that CAH had negatively influenced upbringing, schooling, and relationships. |
| 21 | Meyer-Bahlburg et al (2008) | USA | Cross-sectional comparative study | 143 women with CAH in total (40 SW, 21 SV, 82 NC) vs 24 controls (sisters and female cousins) + 67 diethylstilbestrol-unexposed female controls and 60 diethylstilbestrol -unexposed male controls | Female | 18–61 y | Bisexual and homosexual orientation were more frequent in both classical and NC CAH compared with controls, correlating with the degree of prenatal androgenization (SW > SV > NC > controls). Orientation dimensions were intercorrelated, forming one latent factor predicted by prenatal androgen exposure and masculinized childhood behavior. |
| 23 | Berenbaum et al (2000) | USA | Cross-sectional observational study | 23 CAH girls | Female | 3–12 y | Girls with CAH displayed markedly male-typical toy play and reduced interest in infants. Behavioral masculinization was strongly linked to indicators of prenatal androgen excess (salt-wasting form, early diagnosis, higher Prader score) but unrelated to postnatal androgen levels (17-OHP, bone age, growth rate) |
| 24 | Strandqvist et al (2025) | Sweden | Comparative cross-sectional | 42 women with CAH (C-CAH; n = 29, NC-CAH; n = 13) and 11 women with CAIS vs 147 female and 142 male controls | Female | NC-CAH 30-62 y, C-CAH 26-60 y, CAIS 22-53 y | Women with CAIS and women with NC-CAH responded in a pattern not different from female controls on most questions regarding gendered behavior. Women with C-CAH and women with CAIS responded more similarly to male controls than female controls on the friendship questionnaire. Women with C-CAH worked in occupations with a male sex distribution whereas females with CAIS worked in occupations that were not different from those of female or male controls. More severe forms of CAH were associated with a response pattern more in line with that of male controls, whereas the opposite was true for females with less severe forms of CAH. |
| 25 | Daae et al (2020) | Norway, Sweden, international (multi-center) | Systematic review | 927 CAH assigned female at birth (46, XX) and 274 CAH assigned male at birth (46, XY and 46, XX) | 274 M / 927 F | Not specified (all ages, studies included adults, adolescents, children) | Assigned females at birth (46, XX) with CAH had a greater likelihood to not have an exclusively heterosexual orientation than females from the general population, whereas no assigned males at birth (46, XY or 46, XX) with CAH identified themselves as non-heterosexual. |
| 26 | Asperholm et al (2025) | Sweden | Cross-sectional neuropsychological study | 42 women with CAH (C-CAH; n = 29, NC-CAH; n = 13) and 11 women with CAIS vs 147 female and 142 male controls | Female | NC-CAH 30-62 y, C-CAH 26-60 y, CAIS 22-53 y | C-CAH women had a different cognitive profile from female and male controls. CAIS women were not different from male controls. NC-CAH women had a relative advantage on female-favoring tasks. |
| 27 | Karlsson et al (2017) | Sweden | Observational case–control study | 55 CAH vs 58 controls | 25 M / 30 F | 16–33 y | Adolescents and adults with CAH showed mild but significant impairment in verbal and visuospatial working memory and inhibition vs controls; overall intelligence was normal; impairment associated with null genotype and prenatal dexamethasone; daily function typically unaffected. |
| 28 | Espinosa Reyes et al (2024) | Cuba | Descriptive, cross-sectional, observational study | One female group with CAH (n = 13) and two control groups (one male and one female, n = 13 and n = 13, respectively) | Female | 10–19 y (13.77 ± 3.44 years) | Female adolescents with CAH had worse visual working memory compared to matched controls, but not in verbal memory. Age at diagnosis was negatively associated with the memory tests. |
| 29 | Van’t Westeinde et al (2020) | Sweden | Case-control MRI study with neuropsychological tests | 37 CAH (no prenatal DEX) + 8 CAH (prenatal DEX) patients vs 43 (26 female) healthy controls | 21 F / 16 M (CAH no DEX), 2 F / 6 M (CAH DEX) | 16–33 y | CAH patients, especially those treated prenatally with DEX, had reduced whole brain volume and altered cortical structure, with poorer visuospatial working memory. Structural abnormalities were present regardless of treatment dose, phenotype, or genotype. |
| 30 | Webb et al (2018) | UK | Cross-sectional case-control MRI & cognitive study | 19 CAH (of which, 1 NC) vs 19 controls | Female | 18–50 y | Widespread reductions in white matter structural integrity, reduced working memory, processing speed, and digit span and matrix reasoning scores in CAH patients, despite similar education and intelligence to controls. CAH patients exposed to higher glucocorticoid doses had greater abnormalities in white matter microstructure and cognitive performance. |
| 31 | Messina et al (2020) | Sweden | Observational case-control, neuropsychological study | 43 children / adolescents with CAH (30 SW, 12 SV, 1 NC CAH), of which 11 prenatally DEX vs 52 controls (27 F) | 23 F / 20 M | 7–17 y | Early-diagnosed CAH patients treated with hydrocortisone showed normal cognitive and executive functioning overall. |
| 32 | Falhammar et al (2014) | Sweden | Population-based registry cohort | 253 males with CAH (clinical severity established in 200 patients: 105 SW, 76 SV, 19 NC) vs 25,300 controls | Males | Median age at last observation = 23.2 y (range 0.5–80); SW: 0.5–65.2, SV: 1.4–79, NC: 1.8–49 | Increased psychiatric morbidity in CAH males. Psychiatric morbidity was not raised in the most severe genotype group. Late diagnosis of CAH may explain some of the findings. Those born before the introduction of neonatal screening were more affected, which may be explained by the higher age. |
| 38 | Gunawardana et al (2024) | Multinational | Systematic review and meta-analysis, parent/child reported HRQoL | 781 children in systematic review, 227 in meta-analysis (SW 398, SV 104, NC 73, CLASSIC not specified 203, unknown 3) | 285 M / 495 F (1 not reported) | 0–21 y | Children/adolescents with CAH have similar physical HRQoL to controls, but lower psychosocial HRQoL—especially in school, emotional, and social domains (parent- and self-report). Impaired QoL linked to poor disease control, complications, adherence issues, and older age. |
| 39 | Brener et al (2019) | Israel | Single-center, cross-sectional, case-control study | 23 NCCAH children and adolescents vs controls (6 healthy siblings + controls from the general healthy pediatric population from USA and from Israel) | 7 M / 16 F | 5–18 y | No difference in HRQoL between NCCAH patients and healthy siblings or controls, except for emotional domain in U.S. reference; BMI-SDS positively correlated with school, psychosocial HRQoL scores; patients well controlled anthropometrically. |
| 40 | Strandqvist et al (2014) | Sweden | Population-based registry cohort | 588 CAH patients (SW 240, SV 167, NC 75, unknown 106) | 335 F / 253 M | Patients born 1925–1991 | Patients with the severe forms were more affected by the disease, and women were more affected than men, especially regarding education and fertility aspects. Despite the increased risk for women with SW CAH not to finish primary school, they were more likely to have a high income. All patients and particularly the men were more often on sick leave than controls. Both men and women were more likely to have disability pension. |
| 41 | Collaer et al (2016) | USA | Cross-sectional, cognitive and neuroimaging study | 69 adolescents and adults with CAH (of which, 62 SW and 4 SV) vs 59 controls | 40 F / 29 M | 12–45 y | Spatial perception and quantitative skill were not enhanced (i.e., made more male-typical) in females with CAH. Instead, individuals with CAH, particularly those aged 20 years and older, showed weakened performance in these domains. Also, short-term memory reductions were found in both adolescent and adult patients with CAH, and short-term memory predicted spatial perception and quantitative performance independent of age, sex, and diagnosis effects. |
| 42 | Ferreira et al (2021) | Portugal | Single-center cross-sectional study | 19 NCCAH children vs controls | 13 F / 6 M | 2–18 y (11.9 ± 4.9 y) | Health-related quality of life (HRQoL) scores were generally similar to controls in most age groups, except parents of 8–12-year-olds reported lower psychosocial HRQoL. Self-reported scores from children were not statistically reduced; parent-child perception differed in mid-childhood. |
| 43 | Kung et al (2024) | Hong Kong / UK | Systematic review and multilevel meta-analysis | 20 independent samples of individuals with classic CAH vs controls (aggregate n > 700) | Varied across studies | Varied across studies (childhood to adolescence) | Females with CAH showed strongly male-typical play behaviour compared with control females, whereas males with CAH did not differ from control males. |
| 45 | Berenbaum et al (2018) | USA | Cross-sectional observational study | 54 girls with CAH (40 C, 13 NC) | Females | 10–13 y | Girls with both C-CAH and NC-CAH interacted more with girls than with boys, with no significant group differences. The groups did not differ significantly in gender identity or gender cognitions, but girls with C-CAH spent more time in male-typed activities and less time in female-typed activities than did girls with NC-CAH. |
| 49 | Harasymiw et al (2023) | USA | Retrospective cohort study (health-insurance claims) | 1,626 individuals with CAH (1,056 commercial + 570 Medicaid) vs ~21 million controls | Commercial: approx. 50/50 F/M; Medicaid: data incomplete but includes children, adolescents, young adults | 4–25 y | Children, adolescents, and young adults with CAH in the US were more likely to be diagnosed with a depressive or anxiety disorder and to be prescribed antidepressants as compared to their age and sex matched peers. The likelihood of these conditions increased with age and did not follow the same gender distribution commonly observed in the pediatric and young adult population, with a concentration of cases among males with CAH. |
| 50 | Daae et al (2018) | Norway | Systematic review | 510 males with CAH (the same cohort of 253 was used for two studies, but different aspects were investigated) | Males | Varied across studies (0.5–80 y) | Males with CAH exhibited more problems related to internalizing behaviors (anxiety, depression), impaired QoL in most studies, and varied sexual and reproductive health perceptions. |
